# Natural small-molecule enhancers of autophagy induce autophagic cell death in apoptosis-defective cells

**DOI:** 10.1038/srep05510

**Published:** 2014-07-01

**Authors:** Betty Yuen Kwan Law, Wai Kit Chan, Su Wei Xu, Jing Rong Wang, Li Ping Bai, Liang Liu, Vincent Kam Wai Wong

**Affiliations:** 1 State Key Laboratory of Quality Research in Chinese Medicine, Macau University of Science and Technology, Macau, China

## Abstract

Resistance of cancer cells to chemotherapy is a significant problem in oncology, and the development of sensitising agents or small-molecules with new mechanisms of action to kill these cells is needed. Autophagy is a cellular process responsible for the turnover of misfolded proteins or damaged organelles, and it also recycles nutrients to maintain energy levels for cell survival. In some apoptosis-resistant cancer cells, autophagy can also enhance the efficacy of anti-cancer drugs through autophagy-mediated mechanisms of cell death. Because the modulation of autophagic processes can be therapeutically useful to circumvent chemoresistance and enhance the effects of cancer treatment, the identification of novel autophagic enhancers for use in oncology is highly desirable. Many novel anti-cancer compounds have been isolated from natural products; therefore, we worked to discover natural, anti-cancer small-molecule enhancers of autophagy. Here, we have identified a group of natural alkaloid small-molecules that function as novel autophagic enhancers. These alkaloids, including liensinine, isoliensinine, dauricine and cepharanthine, stimulated AMPK-mTOR dependent induction of autophagy and autophagic cell death in a panel of apoptosis-resistant cells. Taken together, our work provides novel insights into the biological functions, mechanisms and potential therapeutic values of alkaloids for the induction of autophagy.

Autophagy is a cellular degradation process that involves the delivery of cytoplasmic cargos, such as aged proteins, mis-folded proteins or damaged organelles, for lysosomal degradation following sequestration in double-membrane vesicles (autophagosomes). Autophagy occurs at a low basal level in cells, turning over proteins and organelles to maintain homeostasis. However, upon conditions of cellular stress, such as nutrient deprivation, oxidative stress, infection or accumulation of protein aggregates, autophagy begins with membrane isolation and expansion to form autophagosomes that sequester all unwanted cytoplasmic materials. Following fusion of the autophagosome with the lysosome to form an autolysosome, the engulfed materials are degraded to recycle intracellular nutrients and energy^1^. Impairment of autophagy and the age-related decline of autophagic function can lead to the pathogenesis of cancers^2^.

Developing mechanisms to circumvent the common problem of chemoresistance in cancer cells to improve the efficacy of anti-cancer therapies is highly desirable. Autophagy, a process that restores metabolic homeostasis through the catabolic lysis of excessive proteins or injured organelles, is considered a potential target for cancer therapy by way of either its pro-death or pro-survival mechanisms^3^. For example, autophagic dysfunction is associated with DNA damage, chromosome instability^4^, and increased incidence of malignancies^5^. Moreover, enhancers of autophagy may play a protective role in cancer therapy by promoting autophagic cell death in tumours or by augmenting the efficacy of chemotherapeutic agents^6^. Several clinically approved and experimental antitumor agents have been shown to induce autophagy-mediated cell death in various types of cancer cells^7^,^8^. Although autophagy may also promote tumour growth by providing energy to poorly-vascularised cancer cells under hypoxic conditions or nutritional deprivation, autophagy-blocking molecules could be used in combination with chemotherapeutic agents to improve their therapeutic efficacy^7^.

Recently, natural compounds from flavonoids, ginsenosides, naphthoquinones and alkaloids have been found to exhibit anti-cancer effects through the modulation of autophagy. For example, plant flavonoids, such as wogonin and luteolin, have been shown cancer cell death through inhibition of autophagy^9^,^10^,^11^. Ginsenosides such as F2^12^ have also been shown to exhibit anti-cancer effects through the modulation of autophagy. Naphthazarin, a naphthoquinone compound, is a microtubule depolymerising agent that induces cell death by activating apoptosis and autophagy^13^, and plumbagin induces G2-M arrest and autophagic cell death by inhibiting the AKT/mTOR (mammalian target of rapamycin) pathway in breast cancer cells^14^. Alkaloids isolated from plants used in Chinese herbal medicine are an important source for drug discovery^15^. The alkaloid berberine exhibits its anti-cancer effects by inducing autophagic cell death and mitochondrial apoptosis in liver cancers^16^, whereas tetrandrine acts as an enhancer of autophagy that induces early G1 arrest in colon carcinoma cells^17^. Additionally, camptothecin and vinblastine are chemotherapeutic drugs that have been approved for clinical use^18^,^19^,^20^,^21^. Therefore, in this study we set out to identify novel enhancers of autophagy from five primary categories of compounds: flavonoids, flavanols, ginsenosides, naphthoquinone and alkaloids. These compounds may exert putative anti-cancer effects through the modulation of autophagic pathways. Using bioactivity-guided screening of selected compounds isolated from natural products, we have identified a group of alkaloids, including liensinine, isoliensinine, dauricine and cepharanthine, that function as novel inducers of autophagy. Here, we present evidence that isoliensinine, dauricine and cepharanthine induce mTOR-dependent autophagy and autophagic cell death in a panel of apoptosis-resistant cells. Taken together, our work provides novel insights into the autophagic effects of selected alkaloids and their potential uses in anti-tumour therapy.

## Results

### Alkaloid compounds induce formation of GFP-LC3 puncta in multiple cancer cells

An increasing number of studies have identified natural compounds from flavonoids, ginsenosides, naphthoquinones and alkaloids as autophagy modulators with potential therapeutic uses in cancers^9^,^14^,^16^. In the current study, we aimed to identify novel inducers of autophagy from five groups of compounds: the flavonoids, flavanols, ginsenosides, naphthoquinones and alkaloids (Table 1). To verify whether the selected compounds were capable of inducing autophagy, we adopted the HeLa human cervical cancer cell line as a model for autophagy detection because it provided a discrete compartment for accurate immunofluorescence imaging analysis^22^. Previously, we successfully demonstrated the autophagic effect of a triterpenoid compound, saikosaponin-d, using HeLa cells^23^. Here, to determine the optimal concentrations of compounds required for induction of autophagy, we first evaluated the cytotoxicity of each compound. Then, all compounds were evaluated for their ability to induce the formation of GFP-LC3 puncta, a marker of autophagy, using concentrations close to their IC_50_ values. As shown in Table 1, compounds with IC_50_ values greater than 100 μM were tested for induction of autophagy at 50 μM, whereas the other compounds were tested at concentrations ranging from 5 to 25 μM.

**Figure 1:**
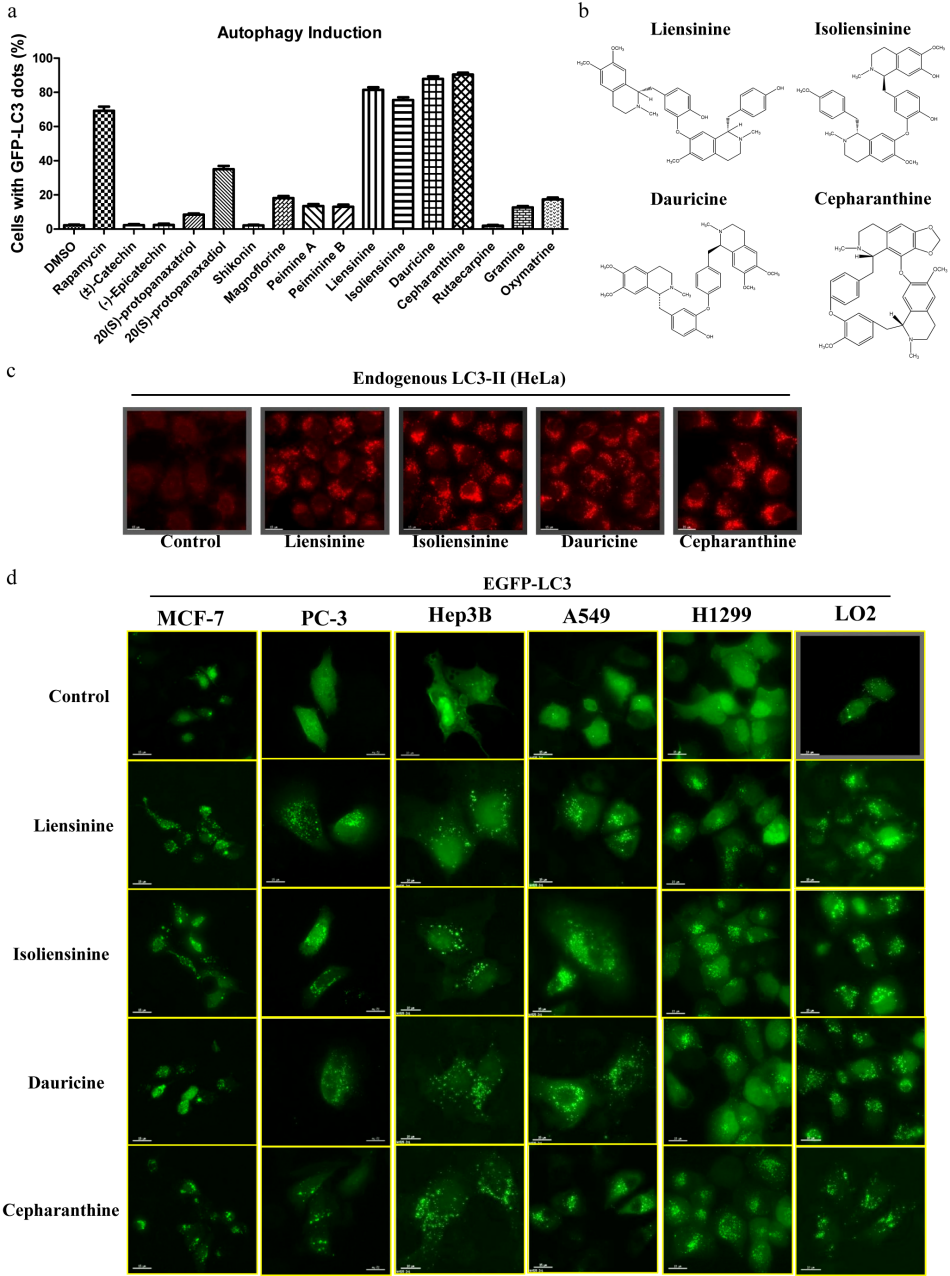
Identification of alkaloid compounds with autophagic activities. a) Detection of GFP-LC3 puncta from compound-mediated autophagy in HeLa cells. Cells were transiently transfected with the EGFP-LC3 plasmid for 24 h and then treated with DMSO (-ve Ctrl), 300 nM rapamycin (+ve Ctrl) or the indicated compounds at their respective IC_50_ concentrations for an additional 24 h. Bar chart represents the quantitation of autophagic cells. The percentages of autophagic cells were calculated as the number of cells with GFP-LC3 puncta (≥10 puncta/cell) divided by the total number of GFP-positive cells in the same field. b) Chemical structures of the four selected alkaloids, liensinine, isoliensinine, dauricine and cepharanthine. c) Endogenous expression of LC3-II in HeLa cells. Cells treated for 24 h with liensinine (20 μM), isoliensinine (10 μM), dauricine (10 μM) or cepharanthine (10 μM) were visualised by fluorescence microscopy after staining with an LC3-II antibody followed by TRITC-conjugated anti-mouse secondary. d) Autophagic effect of alkaloids in various types of cancer and normal cells. MCF-7, PC3, Hep3B, A549, H1299 and LO2 cells were transiently transfected with the EGFP-LC3 plasmid for 24 h and then treated with DMSO (Ctrl), liensinine (20 μM), isoliensinine (10 μM), dauricine (10 μM) or cepharanthine (10 μM) for 24 h. Fluorescence images were captured at 60× magnification; scale bar, 15 μm.

We first transiently transfected HeLa cells with EGFP-LC3 and then incubated them with flavonoid, flavanol, ginsenoside, naphthoquinone or alkaloid compounds for 24 h. Our results indicated that the positive control drug, rapamycin, significantly increased the percentage of cells containing GFP-LC3 puncta, whereas the flavonoid, flavanol ginsenoside, and naphthoquinone compounds induced no or very weak autophagic effects (Fig. 1a). However, a group of alkaloid compounds that included liensinine, isoliensinine, dauricine and cepharanthine (Fig. 1b) demonstrated a marked increase in autophagy induction, as shown by the increased number of cells with GFP-LC3 puncta (Fig. 1a). The formation of LC3-II puncta was further verified by immunofluorescence staining against endogenous LC3-II in HeLa cells (Fig. 1c). In addition, to confirm whether the alkaloid-mediated formation of GFP-LC3 puncta could be induced in other cell types, the cancer cell lines MCF-7, PC-3, Hep3B, A549 and H1299, as well as normal human LO2 hepatocytes, were used to detect GFP-LC3 puncta. As shown in Fig. 1d, 20 μM of liensinine, or 10 μM of isoliensinine, dauricine or cepharanthine, could induce GFP-LC3 puncta formation in both cancer cells and normal hepatocytes, indicating that the autophagic effect of the alkaloids is not cell-type specific.

### The selected alkaloids induce autophagic flux in HeLa cells

**Figure 2:**
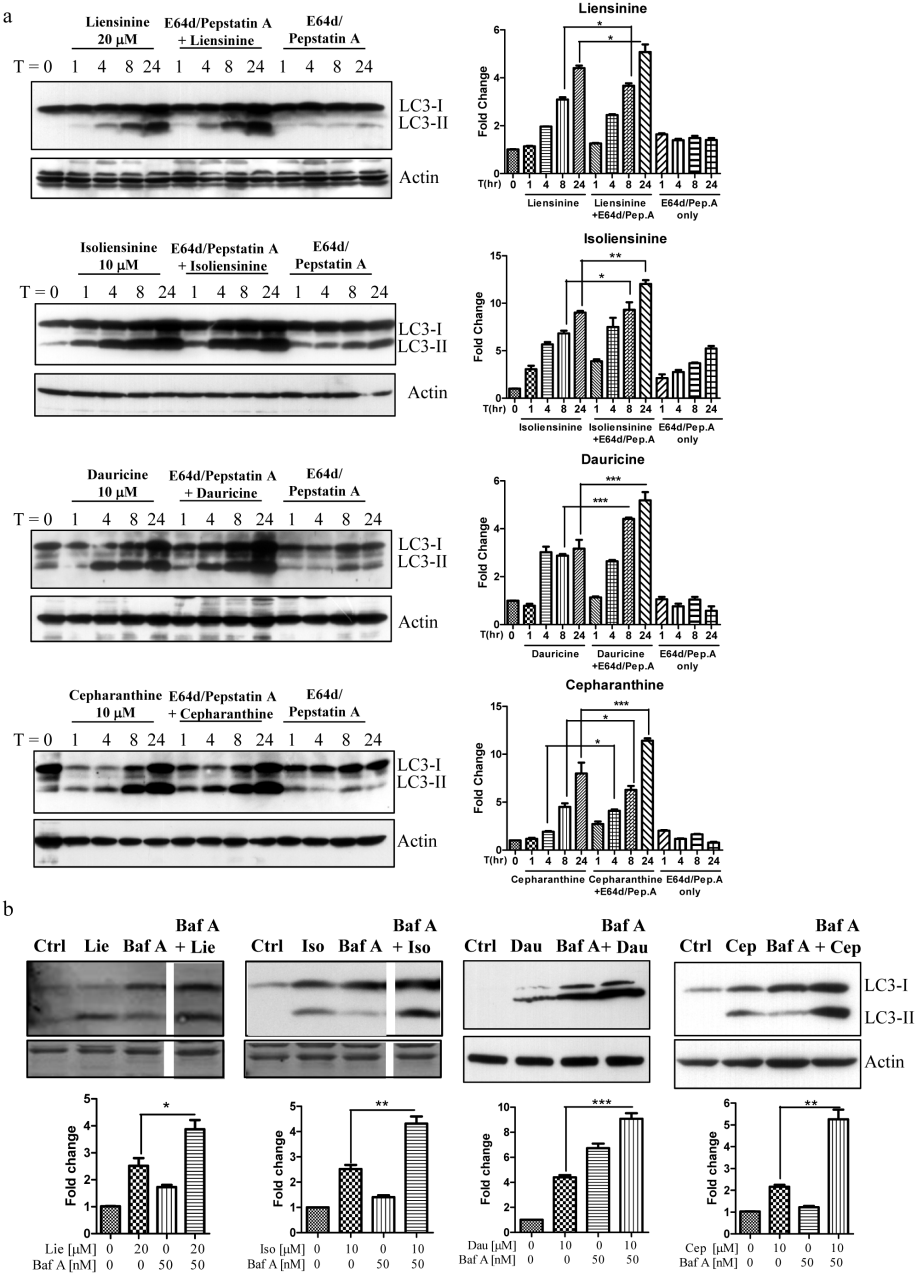
The 4 selected alkaloids induced autophagic flux in HeLa cells. a) Cells were treated with liensinine (20 μM), isoliensinine (10 μM), dauricine (10 μM), or cepharanthine (10 μM) in the presence or absence of 10 μg/mL lysosomal protease inhibitors (E64d and pep. A) for 24 h. b) Cells were treated with liensinine (Lie, 20 μM), isoliensinine (Iso, 10 μM), dauricine (Dau, 10 μM), or cepharanthine (Cep, 10 μM) in the presence or absence of 50 nM lysosomal protease inhibitor, bafilomycin A for 8 h. Cell lysates were analysed by western blot for LC3 conversion (LC3-I, 18 kDa; LC3-II, 16 kDa) and β-actin. LC3-II band intensities were quantified using densitometric analysis and normalised to β-actin. Data are expressed as a fold change relative to the DMSO-treated negative control. Bars are representatives of three independent experiments, and full-length blots are presented in Supplementary Figure 2 & 3. Error bars, S.D. *, P < 0.05; **, P < 0.01; ***, P < 0.001.

It should be noted that the increased formation of autophagosomes, measured by the presence of GFP-LC3 puncta in fluorescence images or LC3 lipidation on a western blot, could have resulted either from the induction of autophagic flux or a failure in fusion of autophagosomes and lysosomes. This could ultimately lead to a reduction in autophagosome turnover^24^. To differentiate between these two possibilities, we measured the conversion of soluble LC3-I to lipid-bound LC3-II in the presence of lysosomal protease inhibitors (E64d and pepstatin A) or bafilomycin A^6^,^25^,^26^. As expected, the four selected alkaloids significantly increased the rate of LC3-II formation in the presence of the inhibitors when compared with the use of either inhibitors or alkaloids alone (Fig. 2). This result suggested that the four alkaloids induced autophagic activity through enhanced autophagic flux and autophagosome formation.

The p62 protein (SQSTM1) is commonly used as a marker to study autophagic flux because it can bind directly to LC3 and then be degraded by autophagy. Thus, the inhibition of autophagy can lead to the accumulation of p62^27^. Unexpectedly, the alkaloid-mediated induction of autophagy was associated with an upregulation of p62, which is a substrate for autophagic degradation (Supplementary Fig. 1a). Real-time PCR analysis revealed that the transcription level of p62 mRNA was also up-regulated after treatment with the alkaloids (Supplementary Fig. 1b). Accordingly, inhibition of protein synthesis by actinomycin D led to a marked reduction of p62 protein in response to selected alkaloid treatments (Supplementary Fig. 1c). These results suggested that p62 was indeed subjected to autophagic degradation upon treatment with the alkaloids.

### Monitoring autophagic flux using mRFP-GFP tandem fluorescent-tagged LC3 (tfLC3)

**Figure 3:**
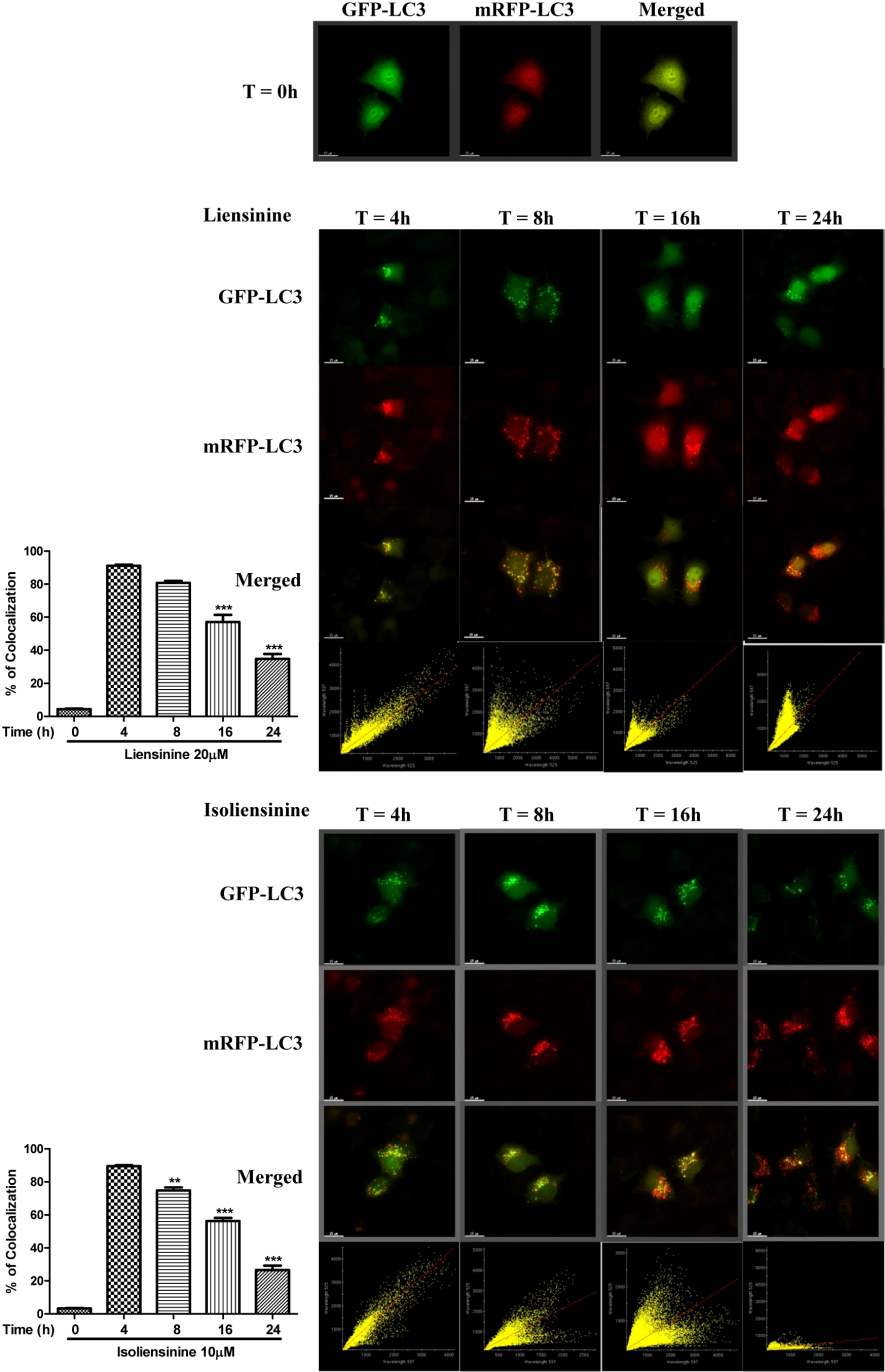
mRFP-GFP-LC3 fluorescence localisation pattern of liensinine and isoliensinine. HeLa cells were transfected with the mRFP-GFP-LC3 plasmids for 24 h. The cells were then treated with liensinine (20 μM) or isoliensinine (10 μM) for 0–24 h. Cells were then subjected to immunocytochemical analysis and mRFP^+^-GFP^+^ (yellow) puncta were scored; scale bar, 15 μm. Each correlation plot is derived from the field shown in the immunofluorescence image. The colocalisation of mRFP with GFP signal from tfLC3 puncta was measured using ImageJ software. The percentage of colocalisation is displayed in the bar chart. The values indicate the average of at least five images. Error bars, S.D. **, P < 0.01; ***, P < 0.001.

**Figure 4:**
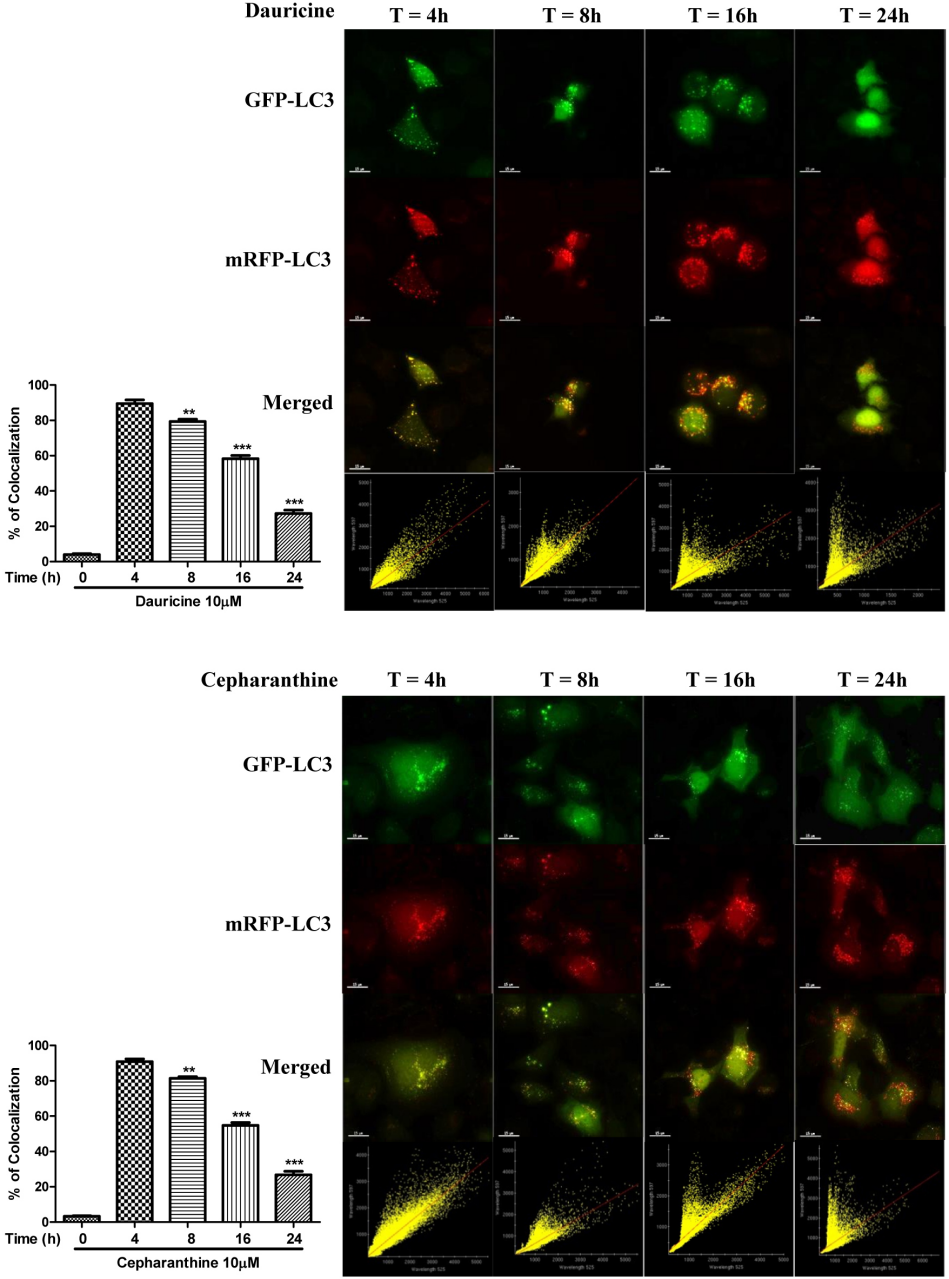
mRFP-GFP-LC3 fluorescence localisation pattern of dauricine and cepharanthine. HeLa cells were transfected with the mRFP-GFP-LC3 plasmids for 24 h. The cells were then treated with dauricine (10 μM) and cepharanthine (10 μM) for 0–24 h. Cells were then subjected to immunocytochemical analysis and mRFP^+^-GFP^+^ (yellow) puncta were scored; scale bar, 15 μm. Each correlation plot is derived from the field shown in the immunofluorescence image. The colocalisation of mRFP with GFP signal from tfLC3 puncta was measured using ImageJ software. The percentage of colocalisation is displayed in the bar chart. The values indicate the average of at least five images. Error bars, S.D. **, P < 0.01; ***, P < 0.001.

Because it exhibits a different localisation pattern from that of GFP-LC3, tfLC3, an LC3 fusion construct with red (mRFP) and green (GFP) fluorescence proteins, is one of the most widely used markers for detecting autophagosomes^28^. Therefore, we used over-expression of tfLC3 to monitor autophagic flux based on the different pH stabilities of GFP and mRFP^29^, as the acidic environment of the lysosome will quench the GFP signal but not the mRFP signal. Therefore, while the yellow merged image (mRFP^+^-GFP^+^) represents the autophagosomes, merged images with red puncta (mRFP^+^-GFP^−^) indicate autophagic flux with the formation of autolysosomes^28^. As shown in Fig. 3 & 4, our results demonstrated a time-dependent decrease in the percentage of cells with mRFP-GFP colocalisation after alkaloid treatments, confirming the induction of autophagic flux by liensinine, isoliensinine, dauricine and cepharanthine in HeLa cells.

**Figure 5:**
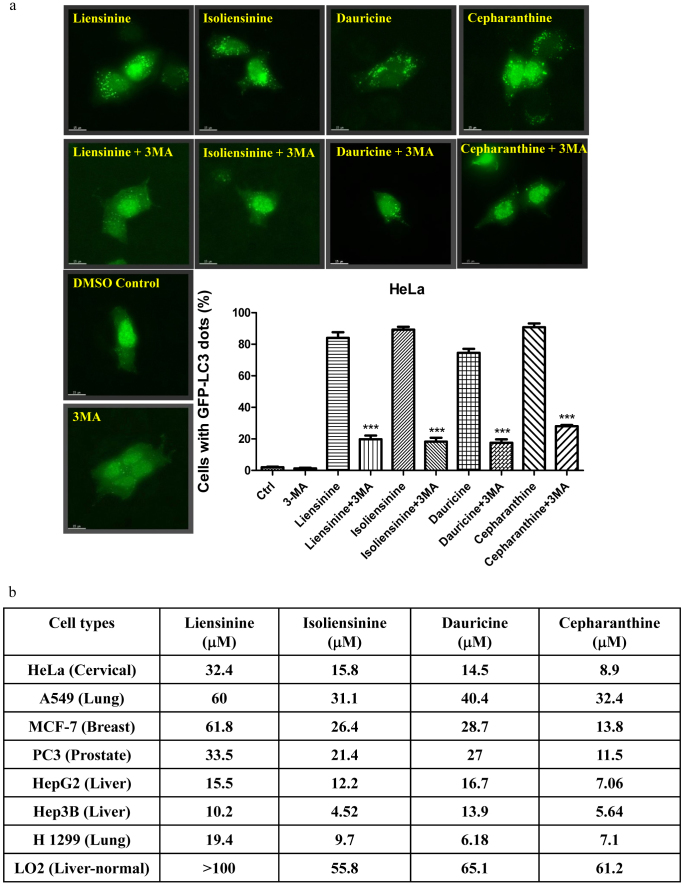
(a) The autophagic inhibitor 3-MA abrogated alkaloid-mediated autophagy. HeLa cells were transiently transfected with the GFP-LC3 plasmid for 24 h and then treated with DMSO (Ctrl), liensinine (20 μM), isoliensinine (10 μM), dauricine (10 μM) or cepharanthine (10 μM), with or without 5 mM of the autophagic inhibitor 3-MA for 24 h. Representative micrographs of cells with GFP-LC3 puncta formation and bar charts with the quantitation of autophagic cells are shown. Percentages of autophagic cells were calculated as the number of cells with GFP-LC3 puncta (≥10 puncta/cell) divided by the total number of GFP-positive cells in the same field. More than 1000 GFP-positive cells were scored for each treatment. Data represent the means of three independent experiments. Error bars, S.D. **, P < 0.01; ***, P < 0.001 for alkaloid-treated cells with and without 3-MA. Fluorescence images were captured at 60× magnification; scale bar, 15 μm. (b) Alkaloid compounds exhibited specific cell cytotoxicity towards a panel of cancer and normal cells. The IC_50_ values shown on the chart are the means of three independent experiments.

Conversely, alkaloid-induced autophagic activity was further validated with 3-methyladenine (3-MA), the well-known PI3K inhibitor commonly used to inhibit autophagy^6^,^30^. Addition of 3-MA prior to alkaloid treatment abrogated alkaloid-mediated autophagy, as shown by the decreased percentage of cells with GFP-LC3 puncta (Fig. 5a). This result further confirmed the autophagy-modulating activity of the alkaloids.

### The alkaloid compounds exhibit specific cytotoxic effect towards cancer cells

Although the alkaloid compounds liensinine, isoliensinine, dauricine and cepharanthine induced autophagy in both cancer and normal cell lines (Fig. 1d), whether the induction of autophagy was toxic to these cells remained unclear. To address whether these alkaloid compounds exhibited specific cytotoxicity to cancer cells, a panel of cancer cells, including HeLa, A549, MCF-7, PC3, HepG2, Hep3B and H1299 were adopted for a cytotoxicity assay. Normal human hepatocytes, LO2, were used for comparison. As shown in Fig. 5b, liensinine, isoliensinine and dauricine were less toxic in A549 lung cancer (mean IC_50_, 31.1–60 μM) and MCF-7 breast cancer cells (mean IC_50_, 26.4–61.8 μM) but showed potent cytotoxicity in HepG2 and Hep3B liver cancer cells (mean IC_50_, 4.52–16.7 μM) and H1299 lung cancer cells (mean IC_50_, 9.7–19.4 μM). As expected, the cytotoxicity of these three compounds was lower in LO2 cells (mean IC_50_, greater than 60 μM), suggesting that they were more potent in cancer cells. With the exception of A549 lung cancer cells, cepharanthine displayed potent cytotoxicity in cancer cells (mean IC_50_, 5.64–13.8 μM) but exhibited fewer cytotoxic effect in normal human hepatocytes (mean IC_50_, 61.2 μM), indicating a higher toxicity of cepharanthine in cancer cells. Thus, these four alkaloid compounds exhibited cytotoxic effects specifically towards cancer cells.

### The alkaloids induce autophagy through activation of the AMPK-mTOR signalling cascade

**Figure 6:**
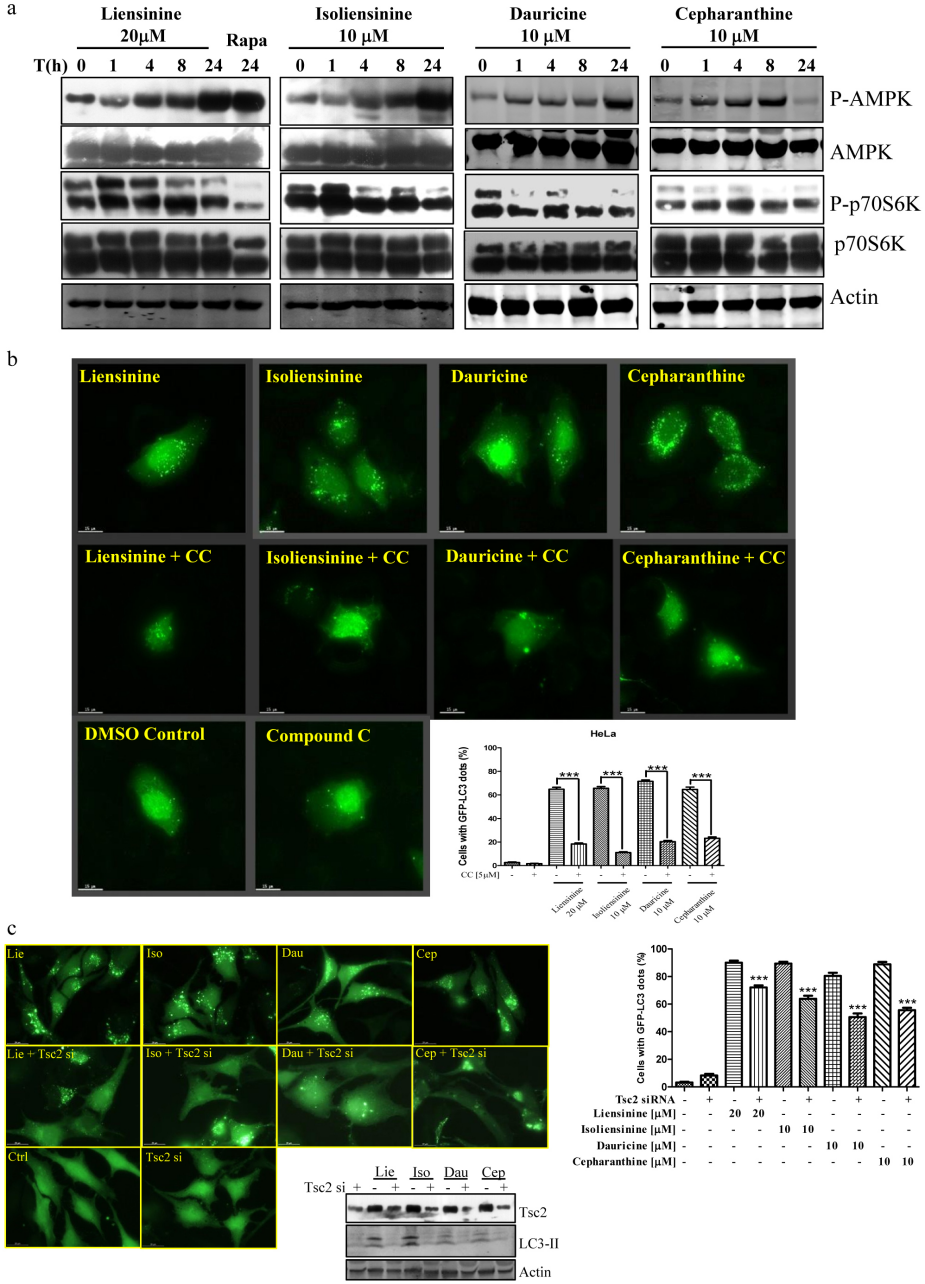
Role of the AMPK-mTOR signalling cascade in alkaloid-mediated autophagy. a) The selected alkaloids activated AMPK-mTOR signalling pathways. HeLa cells were treated with liensinine (20 μM), isoliensinine (10 μM), dauricine (10 μM) or cepharanthine (10 μM) for 0–24 h and analysed for p-AMPK, total AMPK, p-p70S6K, total p70S6K and actin. Rapamycin (Rapa, 300 nM) was used as the positive control. The cropped blots were run under the same experimental conditions, and full-length blots are presented in Supplementary Figure 4. b) An AMPK inhibitor abrogated the alkaloid-mediated autophagic effect in cancer cells. HeLa cells were transiently transfected with the EGFP-LC3 plasmid for 24 h and then treated with DMSO (Ctrl), liensinine (20 μM), isoliensinine (10 μM), dauricine (10 μM) or cepharanthine (10 μM), with or without 5 μM of the AMPK inhibitor compound C (CC), for 24 h. The cells were then fixed for fluorescence imaging, and cells were counted at 60× magnification; scale bar, 15 μm. Bar chart represents the quantitation of autophagic cells with GFP-LC3 puncta. c) TSC2 knockdown abrogated the alkaloid-mediated autophagic effect in cancer cells. GFP-LC3 stable HeLa cells were transfected with or without small interfering RNAs (siRNA) against TSC2 for 48 h. The cells were then treated with DMSO (Ctrl), liensinine (Lie, 20 μM), isoliensinine (Iso, 10 μM), dauricine (Dau, 10 μM) or cepharanthine (Cep, 10 μM) for 24 h. Cells were then fixed for fluorescence microscopy analysis and scoring. Bar chart represents the percentage of cells with GFP-LC3 puncta. In the bottom panel, the TSC2 knockdown cell lysates were also analysed by western blot for the expression of TSC2 and LC3 I/II. Error bars, S.D. ***, P < 0.001. The cropped blots were run under the same experimental conditions, and full-length blots are presented in Supplementary Figure 5.

The AMP-activated serine/threonine protein kinase (AMPK) is a sensor of cellular energy status that is activated under low intracellular ATP conditions such as nutrient deprivation or hypoxia, leading to activation of autophagy through the AMPK-mTOR-dependent pathway^31^,^32^. AMPK directly phosphorylates the tumour suppressing protein tuberous sclerosis complex (TSC2), inducing suppression of mTOR^32^. In *Drosophila*, deletion of TSC2, an inhibitor of TORC1 signalling, blocks autophagy induced by nutrient starvation^33^. Here, our results demonstrated an increase of AMPK phosphorylation in a time-dependent manner following treatment with the alkaloids (Fig. 6a). The AMPK phosphorylation was then accompanied by a reduction in phosphorylated p70S6K, a downstream target of mTOR (Fig. 6a). Furthermore, a significant reduction in the formation of alkaloid-induced GFP-LC3 puncta was also observed in cells pre-treated with the AMPK inhibitor compound C (CC) (Fig. 6b), suggesting the involvement of the AMPK signalling pathway. Using a TSC2 siRNA knockdown experiment (Fig. 6c), we found that there was a decrease in autophagy induction, which was demonstrated by a reduction in GFP-LC3 puncta formation and LC3-II conversion in TSC2-deficient cells. These results further confirmed that the selected alkaloids suppress mTOR activity through the AMPK-TSC2 signalling pathway. Collectively, these four alkaloid compounds induced autophagy via the AMPK-TSC2-mTOR signalling pathway.

### Alkaloid-mediated autophagy promotes cell death

**Figure 7:**
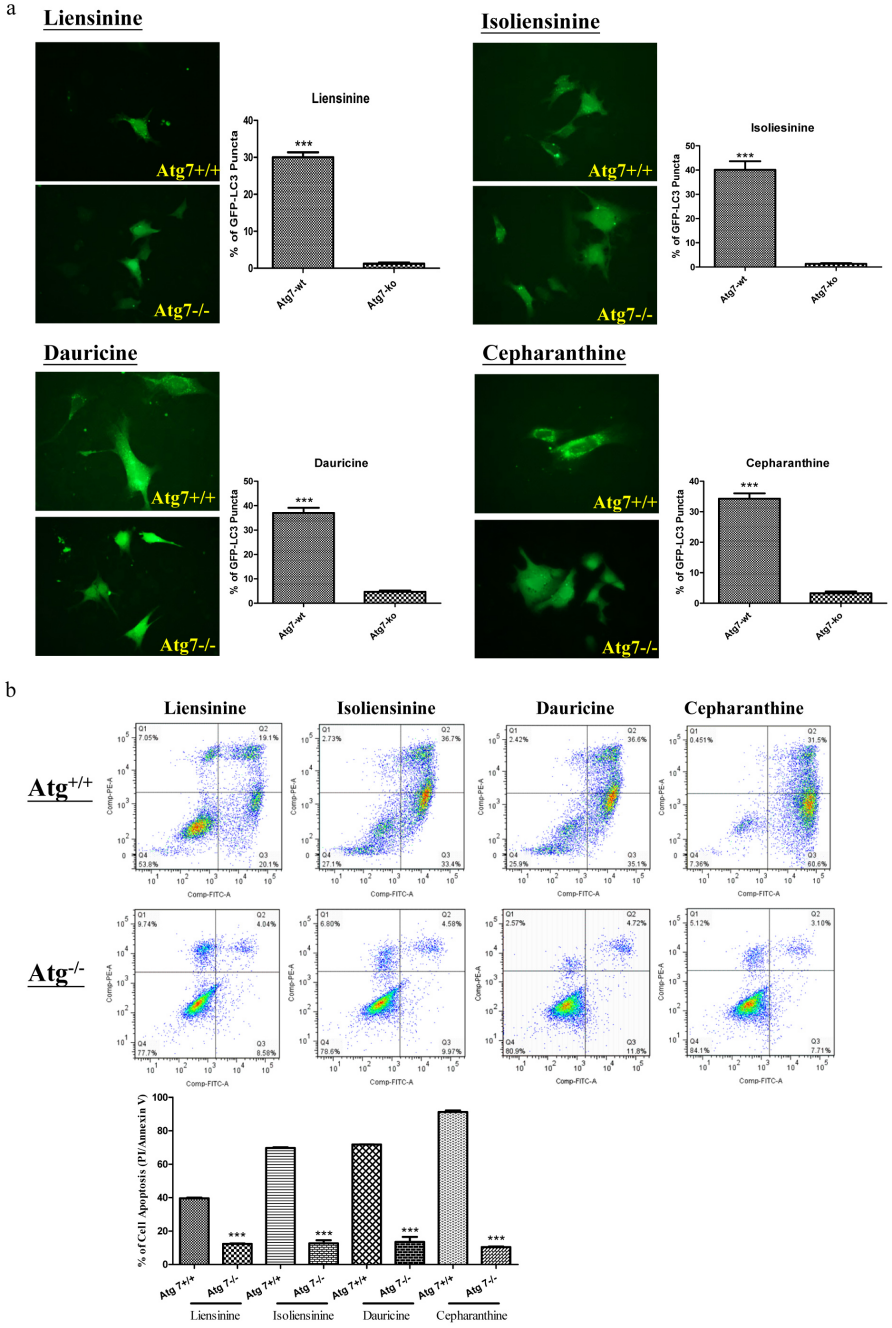
Alkaloid-mediated autophagy and cell death in Atg7-deficient MEFs. a) Both Atg7^+/+^ wild-type and Atg7^−/−^ deficient MEFs were transiently transfected with the EGFP-LC3 plasmid for 24 h and then treated with DMSO (Ctrl), liensinine (20 μM), isoliensinine (10 μM), dauricine (10 μM) or cepharanthine (10 μM) for 24 h. The cells were then fixed for fluorescence imaging and counting. Bar chart represents the quantitation of autophagic cells. Percentages of autophagic cells were calculated by dividing the number of cells with GFP-LC3 puncta (≥10 puncta/cell) by the total number of GFP-positive cells in the same field. ***, P < 0.001, compared to DMSO control. b) Annexin V flow cytometry analysis of the alkaloid compounds in Atg7 wild-type and Atg7^−/−^ deficient MEFs. Both Atg7 wild-type and deficient MEFs were incubated with DMSO (Ctrl), liensinine (20 μM), isoliensinine (10 μM), dauricine (10 μM) or cepharanthine (10 μM) for 24 h. Alkaloid compound-induced cell death in Atg7 wild-type and deficient MEFs was then measured by flow analysis after annexin V staining. Data from the bar chart represents the means ± S.D. of three independent experiments.

Autophagy-related gene 7 (Atg7) is essential for vesicle nucleation and elongation during autophagy^1^. Previous studies demonstrated that Atg7-knockout mice die due to their inability to adapt to neonatal starvation^34^, whereas cancer cells lacking the Atg7 gene fail to response to compound-mediated autophagy^6^,^25^. To investigate whether the four alkaloid compounds used in our study require Atg7 for induction of autophagy, we incubated GFP-LC3 transiently transfected wild-type and Atg7 deficient mouse embryonic fibroblasts (MEFs) with the four alkaloid compounds for 24 h. The alkaloid-treated MEFs were then fixed for quantification of GFP-LC3 puncta formation. As shown in Fig. 7a, 20 μM of liensinine significantly induced formation of GFP-LC3 puncta in Atg7 wild-type MEFs, but not in Atg7 deficient MEFs. Similarly, 10 μM of the alkaloid compounds isoliensinine, dauricine and cepharanthine were found to induce GFP-LC3 puncta formation in Atg7^+/+^ wild-type MEFs only, indicating the involvement of Atg7 in alkaloid-mediated induction of autophagy.

Although many anti-tumour agents can activate autophagy in different types of cancers^7^, it remains controversial whether autophagy promotes cell death or acts as a pro-survival mechanism. Because Atg7^−/−^ deficient MEFs are resistant to induction of autophagy^34^ and because alkaloid-induced autophagy requires Atg7 (Fig. 7a), we used both Atg7 wild-type and Atg7 deficient cells to distinguish whether alkaloid-mediated autophagy leads to cell death or acts as a pro-survival mechanism^6^. To this end, both Atg7 wild-type and Atg7 deficient MEFs were incubated with the alkaloid compounds and then subjected to annexin V flow cytometry analysis. Our results showed that all of the alkaloid compounds exhibited lower toxicity in Atg7^−/−^ deficient MEFs when compared to their wild-type counterparts (Fig. 7b). These data suggest that alkaloid-mediated autophagy would ultimately lead to autophagic cell death, as failed induction of autophagy in Atg7^−/−^ deficient cells markedly abrogated the compound-mediated cytotoxicity. Taken together, our findings suggest that the alkaloid-induced autophagy requires the involvement of Atg7 and that these alkaloid compounds promote cell death through regulation of autophagy.

### Cytotoxic effect of alkaloids in apoptosis-resistant cells

**Figure 8:**
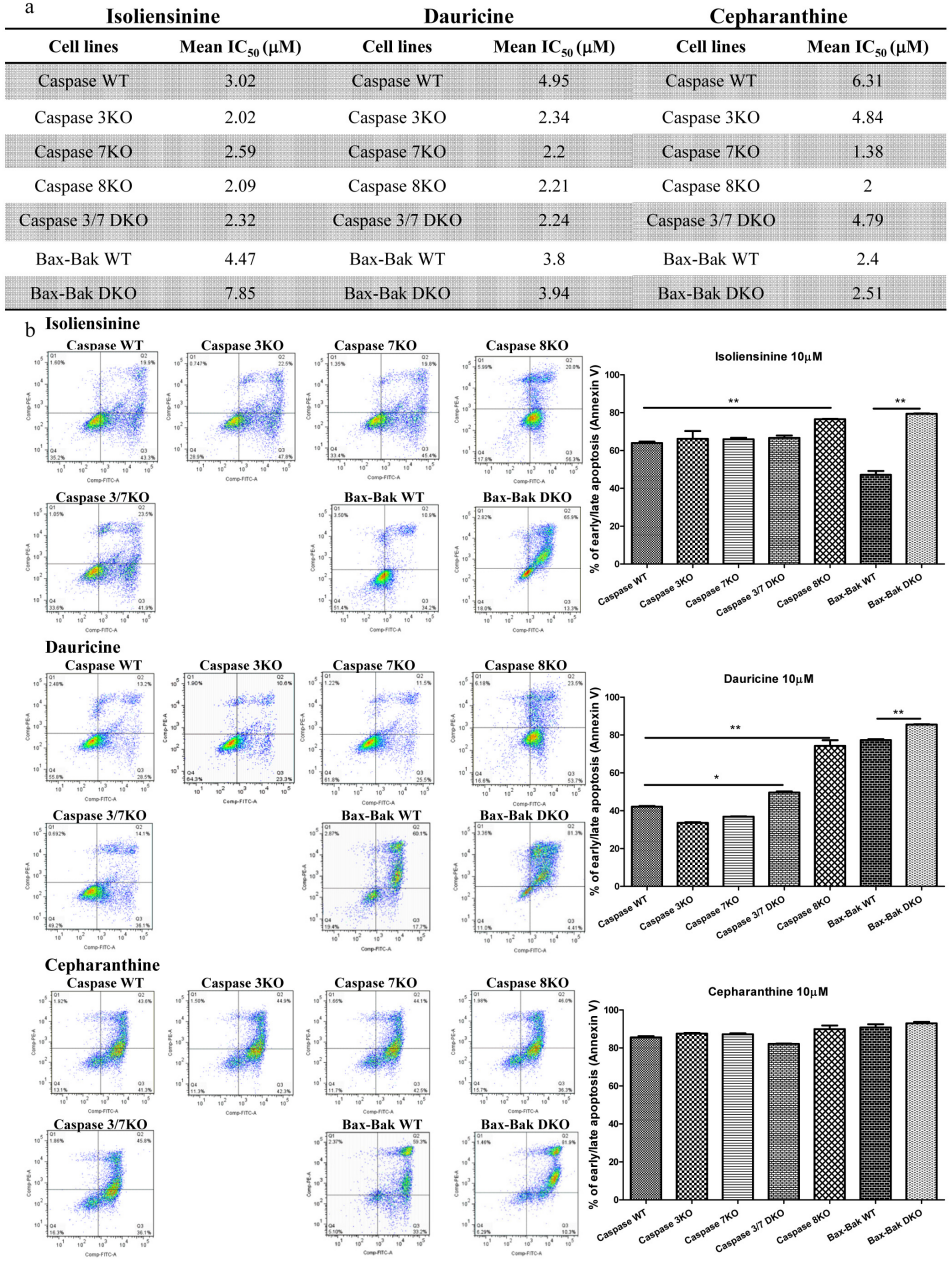
The alkaloid compounds induced autophagic cell death in apoptosis-resistant cells. a) Cytotoxicity of isoliensinine, dauricine and cepharanthine in caspase -3/-7/-8, caspase -3,-7 DKO, Bax-Bak DKO wild-type and deficient MEFs. Both wild-type and deficient MEFs were incubated with the alkaloid compounds at concentrations of 0.19–100 μM for 3 days. Cytotoxicity of the alkaloid compounds in wild-type and deficient MEFs was measured by MTT assay and shown as the mean IC_50_. b) Annexin V flow analysis of the alkaloid compounds in apoptosis-resistant cells. The caspase -3/-7/-8, caspase -3,-7 DKO, Bax-Bak DKO wild-type and deficient MEFs were treated with DMSO (Ctrl), isoliensinine (10 μM), dauricine (10 μM) or cepharanthine (10 μM) for 24 h. Alkaloid compound-induced cell death in these wild-type and deficient MEFs was then measured by flow analysis after annexin V staining. Data from the bar chart represents the means ± S.D. of three independent experiments. ** P < 0.01; * P < 0.05.

Cancer cells are frequently resistant to apoptosis^35^. In light of this, the use of small-molecules to induce autophagic cell death in apoptosis-defective or apoptosis-resistant cancer cells is considered an effective therapeutic approach^36^. To examine whether the newly identified autophagy-inducing alkaloids exhibited potent cytotoxic effects towards apoptosis-resistant cells, we utilised a panel of apoptosis-defective or apoptosis-resistant cells such as caspase 3/-7/-8 deficient MEFs and Bax-Bak double knockout (DKO) MEFs. Among the four identified compounds, isoliensinine, dauricine and cepharanthine were more toxic to HeLa cells and were therefore chosen for the cytotoxicity assays. As shown in Fig. 8a, isoliensinine displayed similar cytotoxicity profiles in both wild-type and caspase -3/-7/-8 deficient, or caspase -3/-7 DKO MEFs. Isoliensinine also showed similar cytotoxic effect towards both Bax-Bak wild-type and DKO MEFs, suggesting that this compound could circumvent the apoptosis-resistant phenotype of cells conferred by genetic deficiencies. On the other hand, both dauricine and cepharanthine displayed higher cytotoxicity in both caspase -3/-7/-8 deficient and caspase -3/-7 DKO MEFs compared with their wild-type counterparts. Furthermore, these two alkaloids also showed similar cytotoxic effects in both Bax-Bak wild-type and deficient MEFs. Dauricine had a mean IC_50_ value of 3.8 μM in Bax-Bak wild-type cells compared to 3.94 μM in Bax-Bak deficient cells, while cepharanthine had mean IC_50_ values of 2.4 and. 2.51 μM, respectively. These results suggest that dauricine and cepharanthine can also circumvent the apoptosis-resistant phenotype of cells caused by various deficiencies in apoptotic genes. Taken together, the alkaloid compounds isoliensinine, dauricine and cepharanthine are potent cytotoxic agents in apoptosis-defective or apoptosis-resistant cells.

To further confirm the above observation, we investigated the cytotoxic effects of these alkaloids in the apoptosis-resistant cells using annexin V flow cytometry analysis. As shown in Fig. 8b, there was coherence between the MTT and flow cytometry data, which suggested that isoliensinine, dauricine and cepharanthine could induce potent cytotoxicity in apoptosis-defective or apoptosis-resistant cells. Therefore, our results highlight the therapeutic potential of developing those active compounds into anti-cancer agents for targeting apoptosis-resistant cancer cells.

## Discussion

The lysosomal degradation pathway of autophagy plays a crucial role in defence against cancers, neurodegenerative disorders, ageing and infections^37^. Accordingly, small-molecules that induce autophagy may have broad therapeutics applications. Our previous study identified the triterpenoid, saikosaponin-d (Ssd), as a novel autophagic inducer that targets apoptosis-resistant cancer cells and has the potential to be developed into an anti-cancer agent^23^,^38^. Here, we further demonstrated that the alkaloid compounds liensinine, isoliensinine, dauricine and cepharanthine are able to induce autophagic-related cell death in cancer cells by inducing autophagy. Therefore, our findings provide insight into the multifunctional role of bioactive alkaloids in inducing death of cancer cells. For instance, rapamycin has been shown to be a potent inducer of autophagy with anti-fungal, immunosuppressive, anti-cancer and neuroprotective properties^39^. As a mammalian target of rapamycin (mTOR) inhibitor, rapamycin and its derivatives CCI-779, RAD001 and AP23573 can effectively inhibit the growth of a broad range of tumours, such as malignant glioma, breast cancer, renal cell carcinoma, non-small-cell lung cancer, mesothelioma, soft-tissue sarcoma, and cervical and uterine cancers^40^. Moreover, recent studies demonstrated that the induction of autophagy by rapamycin is accompanied by an up-regulation of the anti-apoptotic protein Bcl-2, which in turn suppresses activation of autophagy and prevents initiation of programmed cell death^41^. Therefore, Bcl-2 may be an important modulator for balancing the beneficial and detrimental effects of autophagy on cell survival mechanisms.

Through the GFP-LC3 puncta formation assay, which provides a readout of autophagy activation, we identified the alkaloid small-molecules liensinine, isoliensinine, dauricine and cepharanthine, which induce autophagy and lead to conversion of the autophagic marker LC3-I to LC3-II. Although there have been several recent image-based high-throughput screens performed to identify novel autophagic inducers^33^,^42^,^43^, the autophagy-inducing properties of the four alkaloids were not reported in studies using GFP-LC3. Additionally, the role of the newly identified compounds in promoting autophagy in apoptosis-resistant cell lines has not yet been investigated. Here, we showed that induction of autophagy by these alkaloids requires the Atg7 gene. Furthermore, these compounds are capable of inducing autophagy-related cell death, a finding demonstrated by the increased viability of autophagy-deficient cells (Atg7^−/−^ MEFs). Most importantly, the novel autophagic enhancers isoliensinine, dauricine and cepharanthine were further investigated for their potential therapeutic value in the treatment of cancers due to their potent cytotoxicity in apoptosis-resistant cells.

Multidrug-resistance is one of the major causes of therapeutic failure in cancer therapy. In most cases, chemotherapy-resistant cancers have defects in apoptotic pathways. In addition, the mitochondrial/cytochrome c pathway of apoptosis is commonly perturbed in human cancers^7^. For instance, Bax/Bak expression is severely attenuated in many malignancies, and Bax-Bak double-knockout MEFs are resistant to various apoptosis-inducing agents^44^. While caspase-3 and -7 are crucial facilitators of mitochondrial-mediated apoptosis^45^, caspase-3, -8 and -9 have critical roles in apoptosis induced by anti-cancer agents, as well as apoptosis-resistance and drug resistance phenotypes^46^. Although caspase-3 activation has been demonstrated to be required for apoptosis, a recent study revealed the induction of apoptosis in the absence of caspase-3^47^. Moreover, it was demonstrated that ginsenoside (Rh2) induces apoptosis of human hepatoma cells via Bax/Bak and caspase-9/caspase-8 activation^48^. To fully elucidate the cell death mechanism induced by the alkaloids, we investigated the pharmacological action of these molecules using a large spectrum of caspase (-3/-7/-8) and Bax/Bak deficient cell lines. Here, our studies indicated that even when caspase-3/-7/-8 and Bax/Bak genes were deleted, the newly identified autophagy-inducing alkaloid compounds could still activate autophagic cell death independent of the caspases or Bax-Bak. These results point to a potential therapeutic role of the alkaloids in apoptosis-resistant cancers.

In fact, various active compounds isolated from natural products have been found effective in inducing autophagic cell death in apoptosis-resistance cells. For example, Ssd, isolated from *Bupleurum falcatum L*., is capable of inducing autophagic cell death in a panel of apoptosis-resistant cells^23^. Furthermore, ursolic acid, a natural triterpenoid, induces cell death and modulates autophagy in p53 mutant, apoptosis-resistant colorectal cancer cells^49^. Rottlerin, a natural polyphenol purified from the kamala powder, induces autophagic death in caspase-3-deficient cells^50^. Coibamide A, an *N*-methyl-stabilised depsipeptide isolated from a marine cyanobacterium, induces autophagy and cell death in apoptosis-resistant glioblastoma cells^51^.

Among the autophagy enhancers identified in this study, liensinine and isoliensinine belong to the bisbenzylisoquinoline alkaloids, which are isolated from seed embryos of *Nelumbo nucifera* and are known to function as anti-HIV agents^52^. A review of the literature shows that liensinine can antagonise ventricular arrhythmias via suppression of the human ether-a-go-go-related gene (hERG) potassium channel^53^, whereas isoliensinine exhibits potent anti-inflammatory effects in the suppression of bleomycin-induced pulmonary fibrosis^54^. However, recent studies indicated that human breast cancer resistance protein (BCRP) could mediate the excretion of liensinine, thus affecting its pharmacological activity and disposition in cancer cells^55^. These findings may help to explain why liensinine is less potent in cancer cell models.

Dauricine, a bioactive compound isolated from *Asiatic Moonseed Rhizome*, has been commonly used to treat inflammatory diseases in Chinese medicine^56^. Recent studies revealed that dauricine exhibits potent anti-cancer effects. For instance, dauricine was shown to inhibit proliferation and invasion of colon cancer cells, and induce apoptosis by suppression of the NF-kappaB signalling pathway^56^. Accordingly, our discovery of the autophagic effect of dauricine may provide insight into the anti-cancer activities of this compound, particularly in multidrug-resistant and apoptosis-resistant cancer cells.

Cepharanthine is a biscoclaurine alkaloid isolated from the plant *Stephania cepharantha*. It exhibits anti-cancer, anti-inflammatory, anti-allergenic and immunomodulatory activities^57^. Recent studies indicated that cepharanthine could reverse the drug resistance phenotype of cancer cells through the modulation of an ATPase. Moreover, cepharanthine also inhibits the transport activities of the broadly acting multidrug resistance factor ABCC10 (MRP7) and restores the intracellular accumulation of cytotoxic drugs such as paclitaxel^58^. Thus, our findings provide insight into the anti-cancer effects of cepharanthine that result from its induction of autophagy.

While the underlying mechanisms and actions of these newly identified autophagic alkaloids remain to be elucidated, the inhibition of mTOR and regulation of Bcl-2 expression during induction of autophagy, as well as other related mechanisms, should be further investigated. With additional insight into the mechanisms of these compounds, these alkaloids could potentially be developed in the future for use as anti-cancer agents.

## Methods

### Chemicals, plasmids, small interfering RNAs and antibodies

All reagents and chemicals were purchased from Sigma (MO, USA) unless otherwise stated. All screening compounds were purchased from China Chengdu Biotechnology Company Ltd. (Chengdu, China) (>98% purity, HPLC). E64D, pepstatin A and compound C were obtained from Calbiochem (Darmstadt, Germany). The pEGFP-LC3 and mRFP-GFP tandem fluorescent-tagged LC3 (tfLC3) plasmids were gifts from Prof. Tamotsu Yoshimori (Osaka University, Japan). Antibodies against LC3B, TSC2, p-AMPK (Thr172), AMPK, p-p70S6K (Thr389) and p70S6K were purchased from Cell Signalling Technologies Inc. (Beverly, MA). The ZyMax™ TRITC-conjugated anti-mouse secondary antibodies were purchased from Invitrogen (Scotland, UK). Actin and p62 antibodies were purchased from Santa Cruz Biotechnology (Santa Cruz, CA). Small interfering RNAs targeting TSC2 and a non-targeting control were obtained from Qiagen (Hilden, Germany).

### Cell culture

All cells were obtained from the American Type Culture Collection (Rockville, MD) unless otherwise specified. Immortalised wild type and Atg7-deficient mouse embryonic fibroblasts (MEF) were kindly provided by Prof. Masaaki Komatsu (Juntendo University, School of Medicine, Japan). Immortalised wild-type and Caspase 3/7-deficient MEFs were a kind gift from Prof. Richard A. Flavell (Yale University School of Medicine, United States). Immortalised wild type and Caspase 8-deficient MEFs were kindly provided by Prof. Kazuhiro Sakamaki (Kyoto University, Graduate School of Biostudies, Japan). Immortalised wild-type and Bax-Bak double knockout MEFs were kindly provided by Prof. Shigeomi Shimizu (Tokyo Medical and Dental University, Medical Research Institute, Japan). All cells were cultured with medium supplemented with 10% foetal bovine serum (FBS), 50 U/ml penicillin, and 50 μg/ml streptomycin (Invitrogen, Paisley, Scotland, UK). All cell cultures were incubated in a humidified incubator at 37°C with 5% CO_2_.

### Quantification of GFP-LC3 puncta

GFP-LC3 puncta were quantified as described previously^23^. Briefly, cells were fixed with 4% paraformaldehyde (Sigma) and then mounted onto microscope slides with FluorSave™ Reagent (Calbiochem, San Diego, California). Localisation of GFP-LC3 was examined with a Nikon ECLIPSE 80i microscope. Images were captured with a Spot RT3™ digital CCD camera (Diagnostic Instruments, Inc., Melville, NY). To quantify autophagy, the percentage of cells with punctate GFP-LC3 fluorescence was calculated by counting the number of cells showing the punctate pattern of GFP-LC3 and dividing by the total number of GFP-positive cells. A minimum of 1000 cells from randomly selected fields was scored per condition per experiment.

### mRFP-GFP tandem fluorescent-tagged LC3 (tfLC3) immunocytochemistry and fluorescence microscopy

HeLa cells were transfected with mRFP-GFP-LC3 for 24 h. After transfection, the cells were treated with the alkaloids at the indicated concentrations for 0–24 h. Cells were then subjected to immunocytochemistry to measure the efficiency of colocalisation. Each correlation plot is derived from the field shown in the fluorescence microscopic image. Colocalisation of mRFP with GFP in tfLC3 puncta was measured using ImageJ software, and shown as the percentage of the total number of yellow mRFP^+^-GFP^+^ puncta.

### MTT cytotoxicity assays

Compounds were dissolved in DMSO to a final concentration of 100 mM and stored at −20°C until further use. Cell viability was measured using the MTT (3-[4,5-dimethylthiazol-2-yl]-2,5 diphenyl tetrazolium bromide) assay as described previously^22^. The percentage of viable cells was calculated using the following formula: Cell viability (%) = Cells number _treated_/Cells number _DMSO control_ × 100. Data were obtained from three independent experiments.

### Flow cytometry analysis

Cell viability was measured using an annexin V staining kit (BD Biosciences, San Jose, CA, USA). Briefly, cells were treated with the selected alkaloids for 24 h. Cells were then harvested and analysed by multiparametric flow cytometry using FITC-Annexin V and Propidium iodide staining (BD Biosciences, San Jose, CA, USA) according to the manufacturer's instructions. Flow cytometry was then carried out using a FACSCalibur flow cytometer (BD Biosciences, San Jose, CA, USA). Data acquisition and analysis was performed with CellQuest (BD Biosciences, San Jose, CA, USA). Data were obtained from three independent experiments.

### Western blot analysis

Cells were treated with different concentrations of alkaloids (10–20 μM) for 24 h at 37°C. The cell lysates were then harvested and resolved by SDS/PAGE. After electrophoresis, the proteins from SDS/PAGE were electro-transferred to a membrane, which was then blocked with 5% dried milk for 60 min. The membrane was then washed three times for 5 min each with TBST wash buffer and immunoblotted with the appropriate antibodies overnight at 4°C. The membrane was then incubated with HRP-conjugated secondary antibodies for 60 min. Band intensities were quantified with ImageJ (N.I.H.).

### Real time PCR analysis

Cells were incubated with the selected alkaloids for 24 h prior to RNA extraction. Total RNA was extracted from HeLa cells with the FavorPrep™ Total RNA purification mini kit (Favorgen, Ping Tung, Taiwan). cDNAs were synthesised by performing reverse transcription with SuperScript® VILO™ Master Mix (Invitrogen, Grand Island, NY, USA). Real-time PCR was carried out on a ViiA™ 7 Real Time PCR System (Applied Biosystems, Grand Island, NY, USA) using the FS Universal SYBR Green Master Mix (Roche, Indianapolis, IN, USA) according to the manufacturer's instructions. PCR was carried out with the p62 primers 5′-GGA GCA GAT GAG GAA GAT CG-3′ and 5′-GAC GGG TCC ACT TCT TTT GA -3′.

### Statistical analysis

The results were expressed as the means ± SD as indicated. Differences were considered statistically significant when the P-value was less than 0.05. Student's t-test or one-way ANOVA analysis was used for comparison among different groups.

## Author Contributions

B.Y.K.L. and V.K.W.W. designed, carried out the experiments, analyzed the data and prepared the draft of manuscript. W.K.C. and S.W.X. participated the experiments. J.R.W. and L.P.B. provided the small-molecules for drug screening, L.L. and V.K.W.W. conceived the idea, supervised all research and revised the manuscript. All authors reviewed the manuscript.

## Supplementary Material

Supplementary Information Supplementary figures

## Figures and Tables

**Table 1:** Screening of compounds with autophagic activities

Categories	Compounds name	Mean IC_50_ (HeLa)	Dosage used for EGFP-LC3 puncta detection
***Flavonoids***	(±)-Catechin	>100 μM	50 μM
	(-)-Epicatechin	>100 μM	50 μM
***Ginsenosides***	20(*S*)-protopanaxatriol	>100 μM	50 μM
	20(*S*)-protopanaxadiol	22.7 μM	25 μM
***Naphthoquinone***	Shikonin	2.82 μM	5 μM
***Alkaloids***	Magnoflorine	>100 μM	50 μM
	Peimine A	>100 μM	50 μM
	Peiminine B	>100 μM	50 μM
	Rutaecarpine	0.3 μM	5 μM
	Gramine	40.2 μM	50 μM
	Oxymatrine	>100 μM	50 μM
	Liensinine	32.4 μM	20 μM
	Isoliensinine	15.8 μM	10 μM
	Dauricine	14.5 μM	10 μM
	Cepharanthine	8.9 μM	10 μM
