# Supplementary material for: Natural small-molecule enhancers of autophagy induce autophagic cell death in apoptosis-defective cells
Source: Sci Rep. 2014 Jul 1;4:5510. doi: 10.1038/srep05510 (PMC4076737; doi:10.1038/srep05510)

Subject Area: Drug discovery

[*]**Corresponding authors:** Dr. Vincent Kam Wai Wong and Prof. Liang Liu

Address correspondence: State Key Laboratory of Quality Research in Chinese Medicine, Macau University of Science and Technology, Avenida Wai Long, Taipa, Macau, China
Tel: +853-8897 2408, +853-8897 2799; Fax: +853-2882 2799, +853-2882 7222

E-mail address: bowaiwong@gmail.com (VKW Wong); lliu@must.edu.mo (L. Liu)

Natural small-molecule enhancers of autophagy induce autophagic cell death in apoptosis-defective cells

Betty Yuen Kwan Law, Wai Kit Chan, Su Wei Xu, Jing Rong Wang, Li Ping Bai,

Liang Liu[*] and Vincent Kam Wai Wong[*]

State Key Laboratory of Quality Research in Chinese Medicine, Macau University of Science and Technology, Macau, China

Supplementary Figure 1

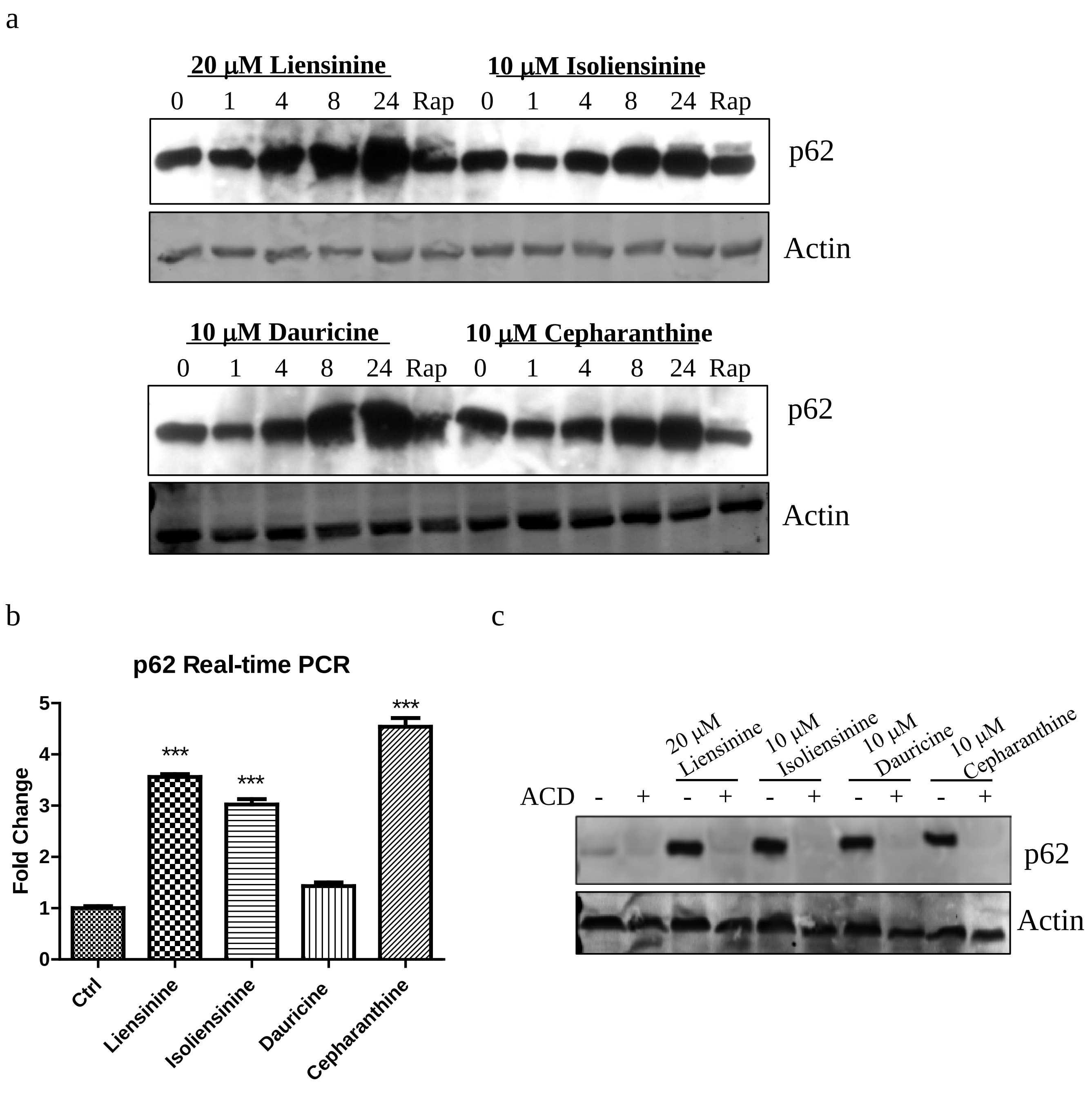

Supplementary Fig. 1. The alkaloid compounds up-regulate the mRNA and protein level of p62. a) Immunoblot of SDS-PAGE showing the protein levels of p62 from 0-24 h of alkaloid treatments at the indicated concentrations. b) Cells were analyzed by real time PCR on p62 mRNA level and normalized against the housekeeping gene (β-actin) after 24 hours of alkaloid treatments at the indicated concentrations. c) Cells were pre-treated with actinomycin D (ACD) (2.5 μg/ml) for 1 h before alkaloid treatments for 0-16 h. Cell lysates were then analyzed for p62 and β-actin. Columns, means of three independent experiments; bars, SEM. **, $P < 0.01$.

Supplementary Figure 2

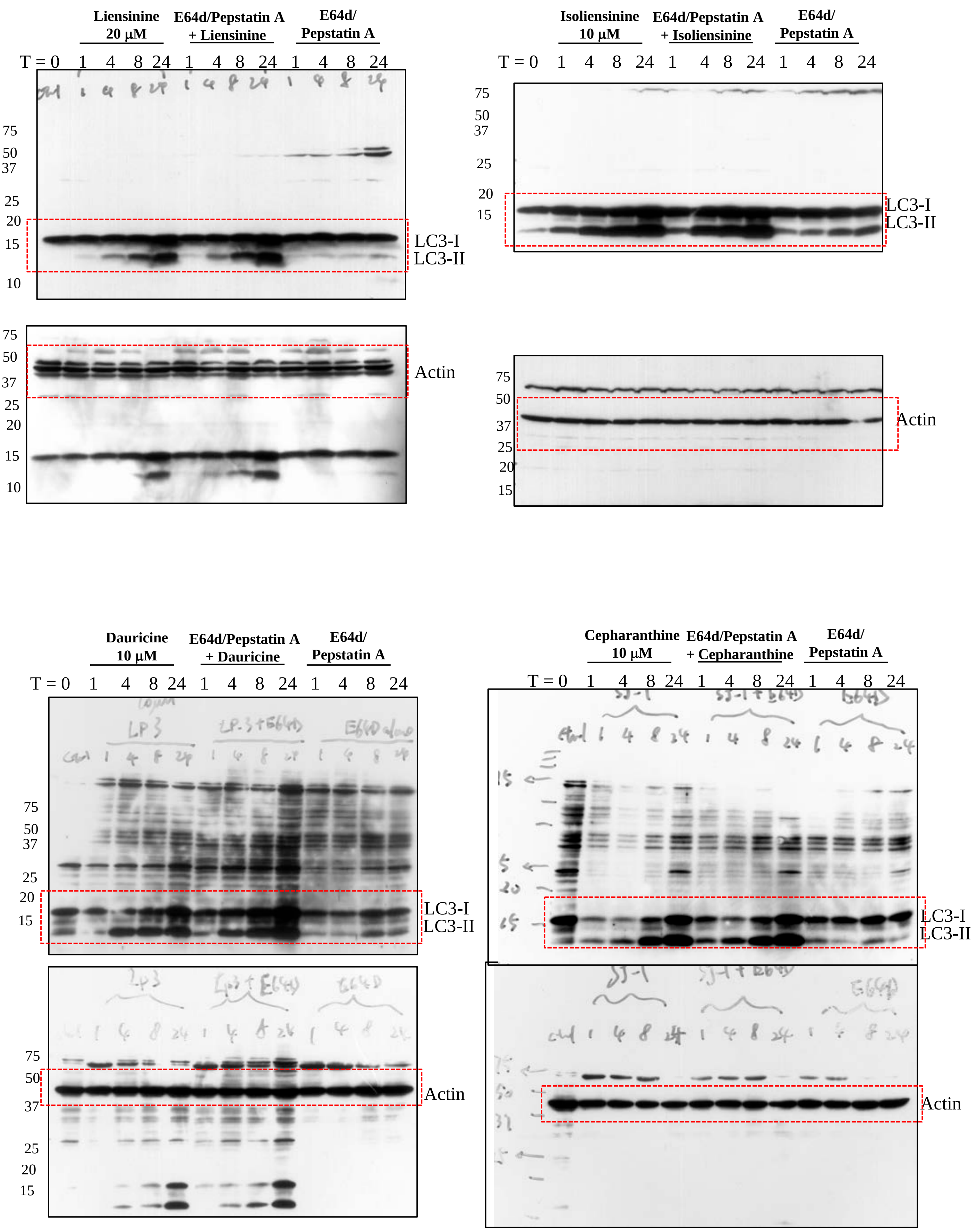

Supplementary Figure 3

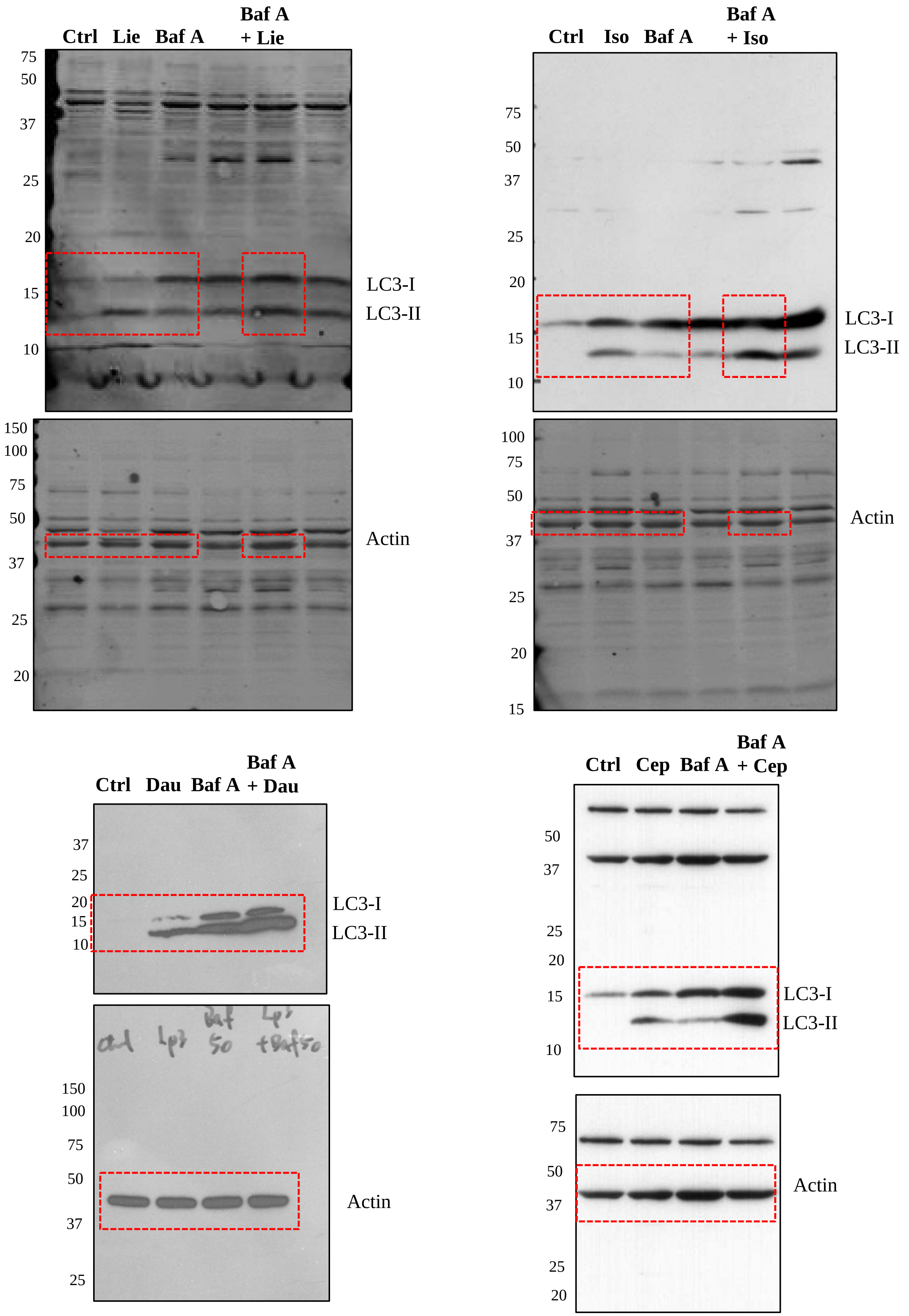

Supplementary Figure 4

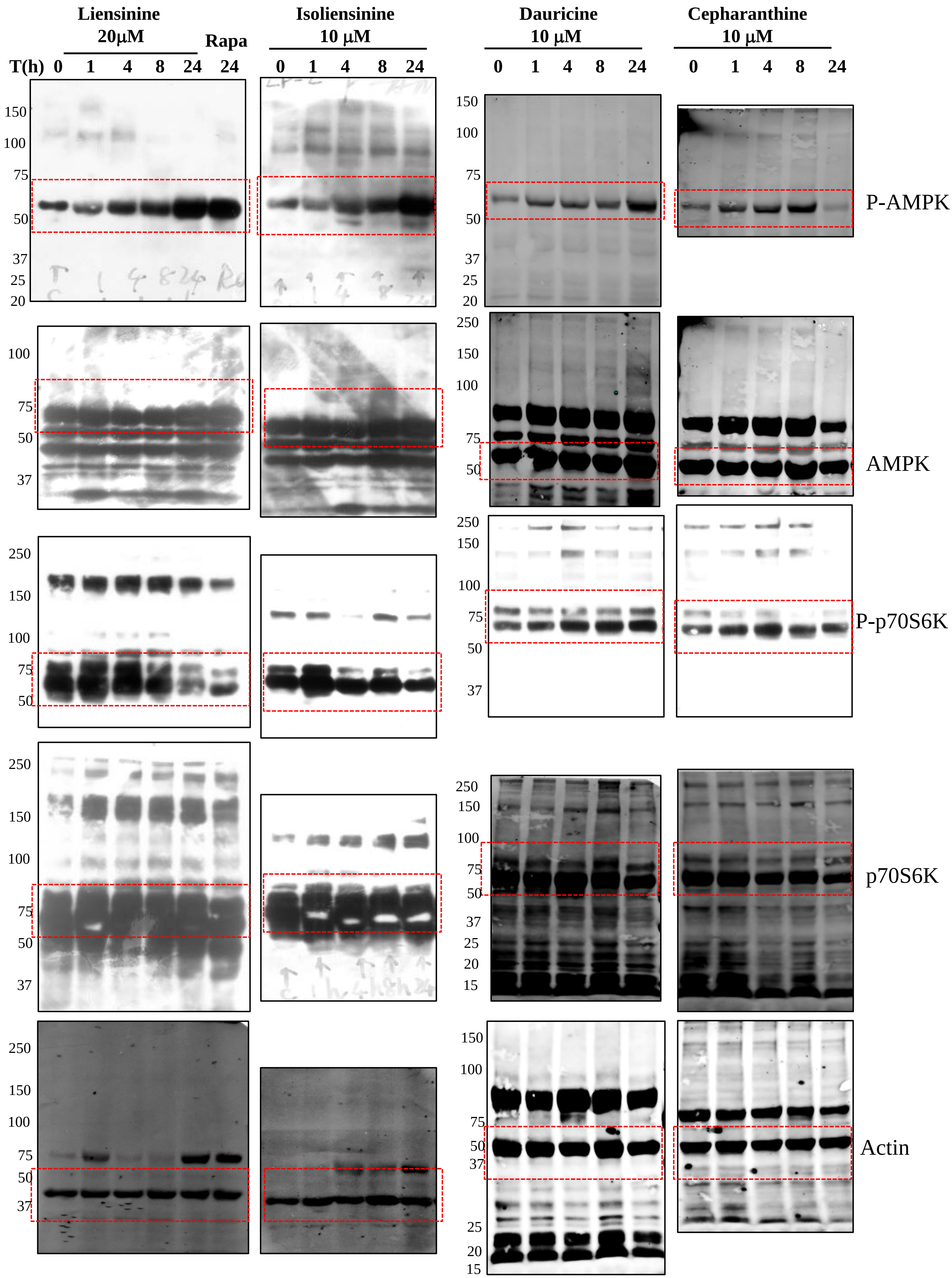

Supplementary Figure 5

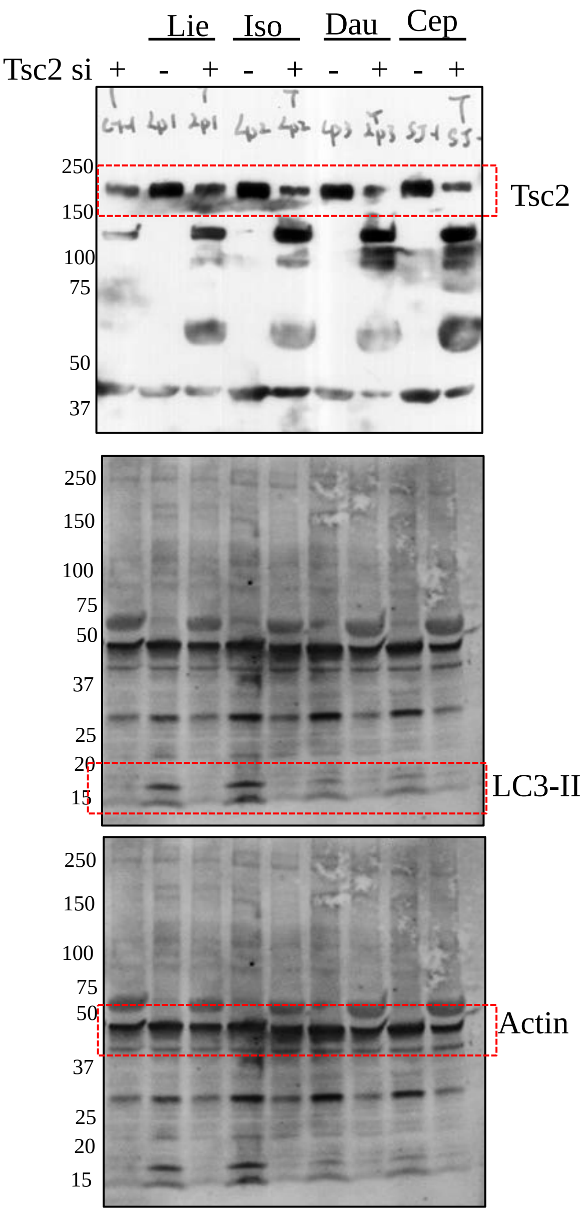

Supplement: Supplementary Information — Supplementary figures [file srep05510-s1.pdf]
